# Elevated urinary excretion of free pyridinoline in Friesian horses suggests a breed-specific increase in collagen degradation

**DOI:** 10.1186/s12917-018-1454-8

**Published:** 2018-04-25

**Authors:** Veronique Saey, Jonathan Tang, Richard Ducatelle, Siska Croubels, Siegrid De Baere, Stijn Schauvliege, Gunther van Loon, Koen Chiers

**Affiliations:** 1 Laboratory of Veterinary Pathology, Department of Pathology, Bacteriology and Avian Diseases, Faculty of Veterinary Medicine, Ghent University, Merelbeke, Belgium; 2 Bioanalytical Facility, Norwich Medical School, University of East Anglia, Norwich Research Park, Norwich, NR4 7UQ UK; 3 Department of Pharmacology, Toxicology and Biochemistry, Faculty of Veterinary Medicine, Ghent University, Merelbeke, Belgium; 4 Deparment of Surgery and anaesthesiology of domestic animals, Faculty of Veterinary Medicine, Ghent University, Merelbeke, Belgium; 5 Department of Large Animal Internal Medicine, Faculty of Veterinary Medicine, Ghent University, Merelbeke, Belgium

**Keywords:** Horse, Aortic rupture, Megaoesophagus, Mass spectrometry, Collagen, Cross-links

## Abstract

**Background:**

Friesian horses are known for their high inbreeding rate resulting in several genetic diseases such as hydrocephaly and dwarfism. This last decade, several studies focused on two other presumed hereditary traits in Friesian horses: megaoesophagus and aortic rupture. The pathogenesis of these diseases remains obscure but an important role of collagen has been hypothesized. The purpose of this study was to examine possible breed-related differences in collagen catabolism. Urinary specimens from Friesian (*n* = 17, median age 10 years old) and Warmblood horses (*n* = 17, median age 10 years old) were assessed for mature collagen cross-links, i.e. pyridinoline (PYD) (=hydroxylysylpyridinoline/HP) and deoxypyridinoline (DPD) (lysylpyridinoline /LP).

Solid-phase extraction was performed, followed by reversed-phase ion-paired liquid chromatography prior to tandem mass spectrometry (MS/MS) detection.

**Results:**

Mean urinary concentrations of free PYD, expressed as fPYD/creatinine ratio, were significantly higher in Friesian horses compared to Warmblood horses (28.5 ± 5.2 versus 22.2 ± 9.6 nmol/mmol, *p* = 0.02) while mean fDPD/creatinine ratios were similar in both horse breeds (3.0 ± 0.7 versus 4.6 ± 3.7 nmol/mmol, *p* = 0.09).

**Conclusions:**

Since DPD is considered a specific bone degradation marker and PYD is more widely distributed in connective tissues, the significant elevation in the mean PYD/DPD ratio in Friesian versus Warmblood horses (9.6 ± 1.6 versus 5.7 ± 1.8, *p* < 0.0001) suggests a soft tissue origin for the increased fPYD levels. Considering that a previous study found no differences in total collagen content between Friesian and Warmblood horses for tendon and aortic tissue, this indicates a higher rate of collagen degradation. The latter might, at least in part, explain the predisposition of Friesians to connective tissue disorders.

## Background

The Friesian horse breed is challenged by several traits in which an important role of collagen has been suggested. In some of these, such as in dwarfism and hydrocephaly, the genetic background has been elucidated. In both diseases, a mutation in a gene involved in protein glycosylation was detected. Dwarfism in Friesian foals, which are also affected by tendon laxity [1], has been attributed to a splice site mutation in B4GALT7 [2]. B4GALT7 has an important role in collagen fibrillinogenesis [3]. A nonsense mutation in B3GALNT2 has been shown to be involved in hydrocephaly in Friesian foals [4]. In humans, mutations in B3GALNT2 are associated with both muscular and brain anomalies [5].

In aortic rupture and megaoesophagus, both presumed to be hereditary traits in Friesians, an increased deposition of clumped collagen at the site of the aortic rupture and in both the dilated and non-dilated part of the oesophagus has been reported [6, 7]. Friesian horses seem to be predisposed for primary gastric rupture as well. It has been suggested that this could also be a manifestation of a presumed underlying soft tissue disorder [8].

Research on collagen metabolism in Friesians is scarce. Our research group has demonstrated an increased area percentage of collagen type I in the thoracic aortic media of Friesians compared to Warmblood horses. This finding suggests the presence of a primary collagen disorder [9]. However, no differences were found in the aortic matrix between Friesian and Warmblood horses using high performance liquid chromatography. In contrast, within the deep digital flexor tendon, Friesians had less lysine hydroxylation and pyrrole cross-linking than Warmblood horses [10].

Mature, trivalent cross-links, called pyridinoline (PYD) or hydroxylysylpyridinoline (HP) and deoxypyridinoline (DPD) or lysylpyridinoline (LP), bind adjacent collagen chains in a triple-helix structure. During tissue degradation, these cross-links are released into the circulation, followed by urinary excretion [11]. In human medicine, excretion of PYD and DPD is mainly monitored to diagnose diseases associated with excessive bone resorption [12, 13].

Not much is known regarding urinary excretion of collagen cross-links in horses. An age-related and diurnal variation, similar as in humans, has been reported [14].

To demonstrate a breed-related change in collagen metabolism, urinary excretion of collagen degradation products (fPYD and fDPD) was measured in Friesian horses and compared to Warmblood horses.

## Methods

### Animals and urine collection

All information regarding the sampled horses is summarized in Table 1. Age, gender, physical exercise, reason for surgery and time of sampling are displayed.

**Table 1:** Overview of animals sampled with information regarding age of horses, gender, regular physical exercise, reason for surgery, time of sampling and measured results

Horse	Age yrs	Gender	Exercise	Surgery	Time hrs	fPYD/creatinine nmol/mmol	fDPD/creatinine nmol/mmol	PYD/ DPD	Horse	Age yrs	Gender	Exercise	Surgery	Time hrs	fPYD/creatinine nmol/mmol	fDPD/creatinine nmol/mmol	PYD/ DPD
Friesian	2	stallion		castration	10	34.8	4.4	8.0	warmbl	6 m	stallion		tendon retraction	9	41.1	17.1	2.4
Friesian	3	mare	yes		17	39.9	4.4	9.0	warmbl	7 m	mare		cecocolic invagination	10	47.4	8.3	5.7
Friesian	4	mare	yes		20	23.4	2.3	10.0	warmbl	3	stallion	yes	castration	9	19.0	4.7	4.0
Friesian	4	mare			17	31.5	2.3	13.7	warmbl	4	mare	yes	sarcoid	10	25.4	4.6	5.5
Friesian	5	mare			18	28.4	3.0	9.6	warmbl	4	mare	yes	Laryngeal hemiplegia	12	20.3	3.5	5.8
Friesian	8	mare	yes		16	26.9	2.7	10.0	warmbl	6	gelding	yes	sarcoid	10	17.1	4.7	3.6
Friesian	8	mare	yes		16	22.9	2.7	8.5	warmbl	6	stallion	yes	castration sarcoid	10	14.1	2.1	6.7
Friesian	9	mare	yes		19	24.9	2.4	10.3	warmbl	7	gelding	yes		13	18.1	2.4	7.4
Friesian	10	mare	yes		11	32.2	3.4	9.4	warmbl	10	gelding	yes	cornea ulcer	11	16.0	2.4	6.7
Friesian	10	mare	yes		16	32.2	3.2	9.9	warmbl	10	gelding	yes		12	16.7	3.0	5.6
Friesian	10	mare	yes		11	32.8	3.1	10.7	warmbl	12	mare		subepiglottis cyst	16	7.1	1.2	6.0
Friesian	11	mare	yes		18	26.0	2.3	11.3	warmbl	12	gelding	yes		12	26.8	2.9	9.1
Friesian	12	mare	yes		11	27.9	2.6	10.9	warmbl	13	gelding	yes		14	19.1	4.2	4.6
Friesian	13	mare	yes		17	26.4	3.6	7.3	warmbl	17	gelding	yes		13	24.0	2.9	8.4
Friesian	15	mare	yes		17	22.5	2.5	8.9	warmbl	18	gelding	yes		15	25.0	7.7	3.3
Friesian	16	mare			17	31.7	3.6	8.8	warmbl	20	mare		urinary bladder tumor	10	18.9	2.9	6.4
Friesian	19	gelding	yes		17	19.6	2.8	6.9	warmbl	20	gelding	yes		14	20.7	3.6	5.8
Median	10				17	27.9	2.8	9.6		10				12	19.1	3.5	5.8

Seventeen Warmblood horses (6 months-20 years old; median age: 10 years old; 5 mares, 3 stallions and 9 geldings) and 17 Friesian horses (2–19 years old; median age: 10 years old; 15 mares, 1 gelding and 1 stallion) were used in this study. Sixteen Friesian and 8 Warmblood horses were sampled once at home during spontaneous urination. The remaining 10 horses (1 Friesian and 9 Warmblood horses) were sampled once during general anesthesia for surgical procedures at the Faculty of Veterinary Medicine of Ghent University, Belgium.

The specimens were immediately frozen. A written consent was obtained from the owner/keeper of each animal. All Friesian horses were purebred and had a pedigree.

The urine was collected in a plastic container and stored at − 20 °C until further analysis (maximum storage time was 9 months). Prior to analysis, samples were defrosted, gently stirred and transferred to 1.5 mL eppendorf tubes.

It is likely that training could have an influence on the pyridinoline cross-linking in bone and possibly in muscle in horses. Both groups of horses however were used for pleasure riding or low level competition.

All samples were collected between September–April, stored and analysed in May of the same year. Time intervals between harvesting and analysis were similar in both horse groups.

### Urine analysis for free pyridinoline (fPYD), deoxypyridinoline (fDPD) and creatinine

Analysis for urinary fPYD and fDPD were conducted following the method described by Tang et al. [15]. Human urine pyridinium cross-links calibrator was used from Immundiagnostik (Bensheim, Germany). In short, 0.5 mL of urine samples pretreated with 0.5 mL hydrochloric acid (40% concentrate) was extracted using solid phase extraction (SPE) column packed with cellulose slurry. Pyridinium cross-links were eluted from the SPE columns and the extracts were transferred into microcentrifuge vials. The eluents were then centrifuged at 10,000 g for 5 min, followed by liquid chromatography and mass spectrometric analysis (LC–MS/MS). The lower limit of quantification was 6 nmol/L for fPYD and 2.5 nmol/L for fDPD.

Urine creatinine was measured to obtain fPYD and fDPD to urine creatinine ratio. A second generation kinetic colorimetric assay was performed, based on the Jaffe method on the COBAS® C501 analyser.^1^ Final results were expressed as nmol/L of fPYD or fDPD per mmol/L creatinine or thus nmol/mmol fPYD or fDPD/creatinine ratio.

### Statistical data analysis

T-tests were performed to compare fPYD/creatinine and fDPD/creatinine urinary ratios between Friesian and Warmblood horses. Significance was set at *p* < 0.05. Commercial statistical software was used (GraphPad Prism, USA).

## Results

**Fig. 1:**
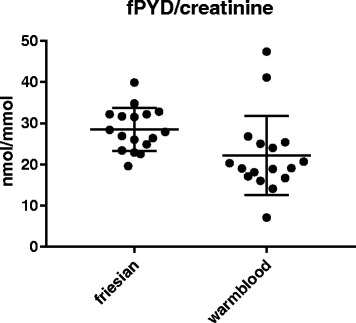
Friesian horses have a significantly higher mean urinary fPYD/creatinine concentration (28.5 ± 5.2 nmol/mmol) (, *n* = 17) in the urine compared to Warmblood horses (22.2 ± 9.6, *n* = 17) (*p* = 0.02)

**Fig. 2:**
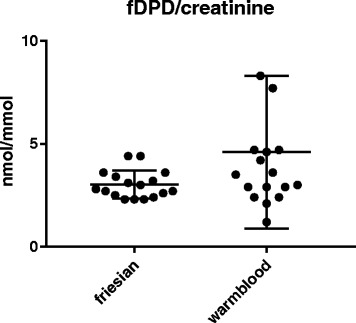
Mean urinary fDPD/creatinine concentrations in Friesian horses (3.0 ± 0.7 nmol/mmol, *n* = 17) showed no significant differences to the values in Warmblood horses (4.6 ± 3.7 nmol/mmol, *n* = 17) (*p* = 0.1)

**Fig. 3:**
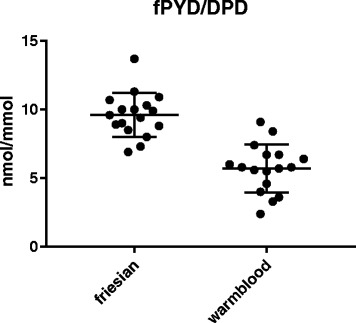
Mean fPYD/fDPD ratios in the urine from Friesian horses (9.6 ± 1.6, *n* = 17) were significantly higher compared to Warmblood horses (5.7 ± 1.8, *n* = 17) (*p* < 0.0001)

The mean urinary fPYD/creatinine ratio was 28.5 (19.6–39.9) ± 5.2 nmol/mmol and 22.2 (7.1–47.4) ± 9.6 nmol/mmol in Friesian and Warmblood horses, respectively (Fig. 1). The mean urinary fDPD/creatinine ratio was 3.0 (2.2–4.4) ± 0.7 and 4.6 (1.2–17.1) ± 3.7 nmol/mmol in Friesian and Warmblood horses, respectively (Fig. 2). The mean urinary fPYD/fDPD ratio was 9.6 (6.9–13.7) ± 1.6 in Friesian and 5.7 (2.4–9.1) ± 1.8 in Warmblood horses (Fig. 3).

There was a significantly higher mean urinary fPYD/creatinine ratio (*p* = 0.02) and fPYD/DPD ratio (*p* < 0.0001) in Friesian versus Warmblood horses. No significant differences were found for mean fDPD/creatinine ratio (*p* = 0.09).

## Discussion

In several mature fibrillar collagen types (I, II, III, V and XI) both PYD and DPD have been detected and their concentration varies considerably between tissues [14]. DPD is mainly present in bone and dentine of type I collagen, while PYD is more widespread and is found in several connective tissues [16]. In the early days, pyridinium cross-links were considered as specific urinary indicators of bone resorption. However, later on, their specificity to bone was questioned. Not only bone and cartilage, but also blood vessels, fascia, muscles and ligaments contain pyridinolines [17].

Urine analysis does not allow to determine the origin of the collagen cross-links. However, considering the low PYD/DPD ratio of bone (3.8 in monkeys) [18] compared to soft tissues, and the mean PYD/DPD ratio of 9.6 in the Friesian horses in this study being very high compared to the result in Warmblood horses (5.8), it is likely that these cross-links originate from soft tissue catabolism. This difference in collagen metabolism could explain the known soft tissue abnormalities in Friesian horses, such as megaoesophagus and aortic rupture. Indeed, a similar elevation in the PYD/DPD ratio has been observed in human patients with scleroderma, a disease characterized by systemic excessive collagen deposition [19] and is attributed to increased collagen catabolism [20]. Furthermore, it was recently shown that deep flexor tendon tissue from Friesian horses contains less PYD cross-links compared to Warmblood horses. Increased collagen catabolism in the tendons from Friesian horses could explain the lower amount of PYD in the tendons [10] and might be related to the increased urinary levels seen in this study. Other connective tissues, such as ligaments, vessels and internal organs, might also have contributed to the high urinary PYD/DPD ratios in Friesian horses.

Besides the origin of collagen cross-links in urine, several other factors might influence their urinary excretion. An age-related variation in PYD and DPD excretion has been reported. The highest amounts of collagen cross-links in urine are found in children during the first weeks and months after birth [21]. The levels of PYD and DPD in babies are 2.5–5 times higher than in infants (5–10 years) and are 15–20-fold compared to adults (20–65 years) [22]. Similar as described in humans, urinary concentrations of PYD and DPD have been shown to be higher in weaned foals (4.6 ± 4 mo) compared to adult horses (16.7 ± 4.6 yrs). These age-related differences are attributed to the ongoing growth with bone remodeling [23]. The mean PYD and DPD/creatinine ratios measured by Black et al. [23] (1999) in these adult (mean age 17 yrs) geldings (six Standardbreds, one Thoroughbred and one Quarter horse) were respectively 15.5 ± 2.0 nmol/mmol and 3.2 ± 0.5 nmol/mmol and were thus comparable to the mean concentrations determined in our Warmblood horse group (22.2 and 4.6 nmol/mmol, median age 10 years). The two youngest horses in this study were Warmblood horses and indeed showed higher fPYD/creatinine levels (41.1 and 47.4 nmol/mmol) and higher fDPD/creatinine levels (17.1 and 8.3 nmol/mmol) compared to the other Warmbloods (mean fPYD/creatinine: 22.2 nmol/mmol and fDPD/creatinine: 4.6 nmol/mmol), which can thus be attributed to bone remodeling.

The gender distribution in the two horse groups was different, as the Friesian horse group contained 15 mares (out of 17 animals) versus 5 mares in the group of Warmblood horses (out of 17 animals). This uneven distribution was due to circumstances beyond our control. To the authors’ knowledge, gender-related differences in the PYD/DPD ratio have not been studied in horses. In humans however, there are no gender differences reported regarding urinary PYD/DPD ratio [22].

Care was taken to include only animals without any signs related to bone or tendon degeneration. For practical reasons, more of the Warmblood horses (52%) were sampled during anesthesia, compared to only 6% of the sampled Friesian horses. Surgical procedures varied from a routine castration to a colic operation. Some Warmblood horses were operated for chronic lesions, such as corneal ulceration or bladder tumor. However, considering the low weight of these organs compared to other tissues such as arteries, tendons and bone, the importance of these tissues in urinary excretion of total collagen is probably insignificant. We have no reason to believe that anesthetic procedures have an influence on urinary excretion of pyridinium cross-links. Alpha 2 adrenergic agonists used during general anesthesia, can increase the volume of urine produced [24]. Therefore, we have normalized the variations in urinary output using the urinary creatinine. Furthermore, we have no reason to believe that anesthetic procedures have an influence on urinary excretion of pyridinium cross-links.

In humans, a well-known circadian variation is present in urinary collagen cross-link excretion. In ante meridiem specimens (AM; 12 h from midnight to noon) both PYD and DPD values are higher compared to post meridiem specimens (PM; 12 h from noon to midnight) [25]. A prominent morning peak has been described in horses as well. In adult horses, PYD and DPD concentrations peak between 2 AM and 8 AM and lowest values are measured from 11 AM to 5 PM and 8 PM to 2 AM. Similar patterns of bone turnover are observed in weaned foals, but they were not significant [23]. The median time of sampling was 12 AM in the Warmblood horses and 5 PM in the Friesian horses. Higher values of fPYD and fDPD could thus be expected in the group of Warmblood horses, based on the fact that they were sampled earlier compared to the Friesians. However, statistically significant lower concentrations of fPYD were found compared to the Friesian horses, which were mainly sampled during the afternoon and evening.

Important to mention is the fact that 3 out of 17 Friesian horses were in foal. Pregnancy is associated with extensive collagen remodeling and can thus have an influence on urinary collagen excretion. During pregnancy, maternal calcium homeostasis is altered in order to meet the fetal demands [26]. Bone resorption occurs in order to obtain sufficient calcium gradients. Bone resorption markers, such as PYD and DPD, increase gradually during pregnancy. The fetal skeleton also contributes to a lesser extent to maternal excretion of cross-links during the last trimester [27]. Mature cross-links such as PYD and DPD have also been shown to decrease and be replaced by immature collagens in the cervix of gravid mice to facilitate compliance and allow birth in the prepartum period [28]. Finally, a peak in urinary PYD levels is established during the puerperium in humans associated with uterine involution [29]. The same increase occurs postpartum in sows [30].

The 3 pregnant Friesian mares in this study were respectively 3, 4 and 5 months in foal. An influence on urinary collagen excretion can thus be expected. However, when these 3 mares were eliminated from the data, mean fPYD/creatinine levels and fDPD/creatinine levels were not that different and even slightly increased. The presence of these 3 pregnant mares in the Friesian horse group did thus not have a significant influence on the mean crosslink levels.

In this study, solely free pyridinium cross-links were analysed. These contribute only a fraction to the total value. However, the correlation between the two forms is strong enough to suggest the free form is representative of the total [31].

## Conclusions

In summary, we have found an elevated PYD/DPD ratio in urine from Friesian horses suggesting an increased collagen catabolism. Considering the high fPYD/creatinine level versus an unaltered fDPD/creatinine level, a soft tissue origin is most likely. An increased collagen catabolism could explain the predisposition of the Friesian horse breed to several soft tissue diseases.

## Abbreviations

AM: Ante meridiem
fDPD: free deoxypyridinoline
fPYD: free pyridinoline
PM: Post meridiem

## Footnotes

1 Roche, Burgess Hill, UK
